# Dilution testing using rapid diagnostic tests in a HIV diagnostic algorithm: a novel alternative for confirmation testing in resource limited settings

**DOI:** 10.1186/s12985-015-0306-4

**Published:** 2015-05-14

**Authors:** Leslie Shanks, M. Ruby Siddiqui, Almaz Abebe, Erwan Piriou, Neil Pearce, Cono Ariti, Johnson Masiga, Libsework Muluneh, Joseph Wazome, Koert Ritmeijer, Derryck Klarkowski

**Affiliations:** Médecins Sans Frontières, Amsterdam, The Netherlands; Médecins Sans Frontières, London, UK; Ethiopian Health and Nutrition Research Institute, Addis Ababa, Ethiopia; London School of Hygiene and Tropical Medicine, London, UK

## Abstract

**Background:**

Current WHO testing guidelines for resource limited settings diagnose HIV on the basis of screening tests without a confirmation test due to cost constraints. This leads to a potential risk of false positive HIV diagnosis. In this paper, we evaluate the dilution test, a novel method for confirmation testing, which is simple, rapid, and low cost. The principle of the dilution test is to alter the sensitivity of a rapid diagnostic test (RDT) by dilution of the sample, in order to screen out the cross reacting antibodies responsible for falsely positive RDT results.

**Methods:**

Participants were recruited from two testing centres in Ethiopia where a tiebreaker algorithm using 3 different RDTs in series is used to diagnose HIV. All samples positive on the initial screening RDT and every 10th negative sample underwent testing with the gold standard and dilution test. Dilution testing was performed using Determine™ rapid diagnostic test at 6 different dilutions. Results were compared to the gold standard of Western Blot; where Western Blot was indeterminate, PCR testing determined the final result.

**Results:**

2895 samples were recruited to the study. 247 were positive for a prevalence of 8.5 % (247/2895). A total of 495 samples underwent dilution testing. The RDT diagnostic algorithm misclassified 18 samples as positive. Dilution at the level of 1/160 was able to correctly identify all these 18 false positives, but at a cost of a single false negative result (sensitivity 99.6 %, 95 % CI 97.8-100; specificity 100 %, 95 % CI: 98.5-100). Concordance between the gold standard and the 1/160 dilution strength was 99.8 %.

**Conclusion:**

This study provides proof of concept for a new, low cost method of confirming HIV diagnosis in resource-limited settings. It has potential for use as a supplementary test in a confirmatory algorithm, whereby double positive RDT results undergo dilution testing, with positive results confirming HIV infection. Negative results require nucleic acid testing to rule out false negative results due to seroconversion or misclassification by the lower sensitivity dilution test. Further research is needed to determine if these results can be replicated in other settings.

**Trial registration:**

ClinicalTrials.gov, NCT01716299.

## Background

The diagnosis of HIV is made on the basis of a reactive screening test or tests followed by a confirmation test. However due to issues of cost, the WHO currently recommends that confirmation testing is not performed in resource limited settings, and instead that diagnosis be made on the basis of an algorithm employing 2–3 rapid diagnostic tests (RDTs) [1]. This strategy has allowed life-saving scale up of HIV diagnosis, as it permits testing to be decentralized outside of the laboratory. The compromise is that without a confirmation test, some individuals will be falsely diagnosed as HIV positive. This risk of false positive HIV diagnosis on the basis of 2 RDT positive results has been shown in a number of settings with different RDTs [2–5]. The risk is increased in lower prevalence populations. The mechanism causing false positive reactions on serological tests is that of non-HIV antibodies cross-reacting with the test antigens [6]. Given the consequences for individuals in terms of the psychological impact, effect on family and community, and possible health consequences of unnecessary exposure to antiretroviral drugs, our group has called for implementation of routine confirmation testing [3]. However, the gold standard for confirmation of HIV testing has been Western Blot (WB) or indirect immunofluorescence assay (IFA) neither of which is suitable for use in peripheral laboratories. Traditional confirmation tests also have limitations in identifying recent seroconversion, can give indeterminate results, and do not allow discrimination between HIV 1 & 2 infections. These limitations have led the US to introduce new guidelines that employ a supplementary testing algorithm rather than a single confirmation test [7, 8]. Samples repeatedly positive on screening assays, are given a supplementary test to confirm infection, and if negative go on to nucleic acid testing (NAT) to rule out a false negative result due to early seroconversion. The only supplementary test currently approved by the FDA is Bio-Rad Multispot HIV-1/HIV-2 Rapid Test. It is a single use flow-through rapid test that yields a result in 15 min., and is able to discriminate between HIV 1 & 2. Bio-Rad Geenius HIV 1/2 Confirmatory Assay, is another single use rapid test that is being evaluated as a supplementary test but is not yet approved for this use by the FDA. We are not aware of any published evaluations done outside of reference laboratories of either test and current pricing limits their use in resource-limited settings. We use a low cost confirmation test which we have shown is feasible for use in a peripheral laboratory, the Orgenics Immunocomb® II, HIV 1&2 Combfirm or OIC. [3]. However this test is not CE marked nor has it been evaluated by WHO. There is therefore an urgent need to develop new confirmation tests or methodologies that are low cost and feasible for use in resource-limited settings.

In this paper, we describe a new methodology to confirm positive HIV screening tests, using serial sample dilution of RDTs. We postulate that by decreasing the sensitivity of the HIV RDT through serially diluting the sample, it will be possible to screen out false positive samples, and correctly identify true positives.

The work reported here formed part of a larger study on HIV diagnostics [9]. In Ethiopia, a tiebreaker regimen consisting of 3 RDTs in series is the national algorithm. HIV (1 + 2) Antibody Colloidal Gold (KHB, Shanghai Kehua Bio-engineering Co Ltd, China) is used as a screening test, followed by HIV 1/2 STAT-PAK® (Chembio Diagnostics, USA) if positive. Where the result of STAT-PAK® is discordant with KHB, a third test, Unigold HIV (Trinity Biotech, Ireland), is used as a tiebreaker to determine the result. Médecins Sans Frontières-Holland (MSF), in response to reported cases of false positive results [10], uses an alternate algorithm in Ethiopia, consisting of two RDTs (KHB, STAT-PAK®) in series, followed by the OIC confirmation test. The primary objective of the study was to evaluate the performance of these two algorithms. Secondary objectives, in addition to evaluating dilution testing, were to evaluate the positive predictive value of weakly reactive RDT test lines [9] and to determine if visceral leishmaniasis (VL), a disease endemic in the region, was associated with an increased risk of false positive reactions. In this paper we focus on the dilution testing objective.

## Methodology

### Setting

The study was conducted in 2 sites in north-western Ethiopia: a MSF supported health centre in Abdurafi, Amhara Region and a hospital in Humera, Tigray Region. The populations included residents as well as high numbers of migrant workers who were present seasonally.

### Inclusion criteria

All clients, aged > = 5 years, presenting to be tested for HIV in the study sites were offered participation in the study. All study participants underwent informed consent procedures and had a written consent form signed by the participant or the guardian.

### Sample size

A sample size of 200 KHB positive and 200 KHB negative participants was chosen based on the WHO guidance for evaluation of RDTs [11]. To achieve the sample, all KHB positive samples were included along with every 10th KHB negative sample until a minimum of 200 positive samples was reached. In order to increase power for the secondary objective on VL, additional participants were recruited until a total of 90 individuals with VL were included. Dilution testing was performed on the total sample set.

### Testing

Initial testing was done at the Counselling and Testing (CT) centres. Laboratory technicians, blinded to the CT results, re- tested each sample on plasma with the 3 RDTs in the national algorithm (KHB, STAT-PAK® and Unigold™). Tests were performed according to manufacturer’s instructions and invalid tests discarded and repeated on new test devices. All samples underwent testing by Western Blot (WB) with technicians blinded to earlier results using MP Diagnostics HIV Blot 2.2. Interpretation of results was based on American Red Cross recommendations [12].

Samples indeterminate on WB were repeated and if still indeterminate, underwent DNA PCR examination using Roche Amplicor DNA v1.5 on dry blood spots (DBS) at the Ethiopian Health and Nutrition Research Institute (EHNRI) laboratory based in Addis Ababa. Global Clinical and Viral Laboratory in South Africa provided quality control for PCR by repeating the samples to confirm results using the Cobas AmpliPrep/Cobas TaqMan HIV-1 Qual test. Samples were sent as dry blood spots (DBS) by international courier, packaged in accordance with IATA regulations for such samples. Where results between the two labs were discrepant, the result from South Africa was used.

The final gold standard result was that of Western Blot, and where Western Blot was indeterminate, the PCR result.

### Dilution technique

Patient plasma was diluted with seronegative plasma from healthy blood donors using a micropipette and microtitration plate. Plasma was defined as seronegative when negative on 2 RDTs and on WB. 10 μL of patient plasma was first diluted 1:10 in 90 μL of negative plasma. This was followed by a serial 4-fold dilution from 1:10 to 1:10,240 in negative plasma.

Testing was performed on Determine™ HIV-1/2 (Alere Laboratories, Japan) using 50 L of diluted sample and read within 15 minutes following manufacturer’s instructions. Tests were interpreted as positive if there was any colouration of the test line. The highest dilution that gave a positive result was recorded. Where the lowest dilution (1/10) was negative, the sample was reported as negative. Invalid tests, where the control line did not appear, were discarded and repeated on a new test device according to manufacturers’ instructions.

### Quality control

All staff underwent training on the study standard operating procedures by the MSF laboratory supervisor, and received regular monitoring and supervision.

### Analysis

Predictive values and sensitivity and specificity were estimated from the 2 × 2 table of observed results after weighting based on the sampling proportion of the KHB positive and negative samples. Confidence intervals for each of the test parameters were calculated using exact binomial intervals.

The proportion of total specimens with the same classification measured concordance between the gold standard and experimental methodology. The kappa statistic was calculated to assess agreement. Statistical analysis was done using Stata version 12 (StataCorp, College Station, Texas, USA).

### Ethical review

The study received approval from the MSF Ethics Review Board, the Ethiopian Health and Nutrition Research Institute Research and Ethical Clearance Committee, and the National Research Ethics Review Committee, Ministry of Science and Technology in Ethiopia.

## Results

2897 individuals were recruited to the study. One sample was excluded due to missing WB/PCR results, and a duplicate was dropped, giving a total of 2895 in the final analysis. 495 were selected for testing by dilution and with the gold standard. 247 were positive on the gold standard for a prevalence of 8.5 % (247/2895).

The serial KHB/STAT-PAK® algorithm correctly identified 247 positives, yielded one false positive and no false negatives for a sensitivity of 100 % (95 % CI: 98.5-100), specificity of 100 % (95 % CI: 99.8-100), negative predictive value (NPV) of 100 % (95 % CI: 98.5-100) and positive predictive value (PPV) of 99.6 % (95 % CI: 97.8-100).

The tiebreaker KHB/STAT-PAK® /Unigold™ algorithm correctly identified 247 positives, yielded 18 false positives, and no false negatives for a sensitivity of 100 % (95 % CI: 98.5-100), specificity of 99.3 % (95 % CI: 98.9-99.6), NPV of 100 % (95 % CI: 98.4-100) and PPV of 93.2 % (95 % CI: 89.5-95.9).

### Results of dilution testing

Results for the performance of each dilution can be found in Tables 1–2. The 1/40 dilution correctly identified 247 positives and 246 negatives, and had two false positives. The 1/160 dilution correctly identified 246 positives and 248 negatives with no false positives, one false negative, while the 1/640 dilution had no false positives and 3 false negatives.

**Table 1:** Results of the dilution test compared to gold standard (N = 495)

Dilution	Result	Number	Number with gold standard result		Concordance	Kappa value*
			Positive (%)	Negative (%)		
1/10	Positive	259	247 (95.3)	12 (4.6)	97.6	0.952
	Negative	236	0	236 (100)		
1/40	Positive	249	247 (99.2)	2 (0.8)	99.6	0.992
	Negative	246	0	246 (100)		
1/160	Positive	246	246 (100)	0	99.8	0.996
	Negative	249	1 (0.4)	248 (99.6)		
1/640	Positive	244	244 (100)	0	99.4	0.988
	Negative	251	3 (1.2)	248 (98.8)		
1/2560	Positive	240	240 (100)	0	98.6	0.972
	Negative	255	7 (2.8)	248 (97.2)		
1/10,240	Positive	221	221 (100)	0	94.8	0.895
	Negative	274	26 (9.5)	248 (90.5)		
TOTAL		495	247 (49.9)	248 (50.1)		

*All p values for the kappa statistics are <0.001

**Table 2:** Performance characteristics of the dilution test compared to gold standard (N = 2895)

Dilution	Sensitivity (95 % CI)	Specificity (95 % CI)	PPV (95 % CI)	NPV (95 % CI)
1/10	100 % (98.5-100)	96.3 % (95.5-97.0)	71.6 % (66.5-76.3)	99.6 % (98.5-100)
1/40	100 % (98.5-100)	99.5 % (99.2-99.7)	95.1 % (91.6-97.3)	100 % (98.5-100)
1/160	99.6 % (97.8-100)	100 % (98.5-100)	100 % (98.5-100)	100 % (99.8-100)
1/640	98.8 % (96.5-99.8)	100 % (98.5-100)	100 % (98.5-100)	99.9 % (99.7-100)
1/2560	97.2 % (94.3-98.9)	100 % (98.5-100)	100 % (98.5-100)	99.7 % (99.5-99.9)
1/10,240	89.5 % (85.0-93.0)	100 % (98.5-100)	100 % (98.3-100)	99.0 % (98.6-99.4)

The sensitivity of the 1/160 dilution was 99.6 % (95 % CI: 97.8-100) with a NPV of 100 % (95 % CI: 99.8-100) and a specificity and PPV of 100 % (95 % CI: 98.5-100).

Concordance between the dilution test and the gold standard of WB resolved by PCR is shown in Table 1. Concordance of the best performing dilution, 1/160, with WB alone was 87.15 % (kappa = 0.771). Excluding the 64 WB indeterminate results gave 100 % concordance (kappa = 1.0).

Incorporating the dilution tests into the tiebreaker algorithm improved the specificity and PPV at the lower dilutions of 1/10 and 1/40. Complete results are shown in Tables 3–4.

**Table 3:** Results of KHB/STAT-PAK®/Unigold™ tiebreaker algorithm with dilution as a supplementary test compared to gold standard (N = 495)

Algorithm	Result	Number	Number with gold standard result		Concordance	Kappa value*
			Positive (%)	Negative (%)		
1/10	Positive	251	247 (98.4)	4 (1.6)	99.2 %	0.984
	Negative	244	0	244 (100)		
1/40	Positive	248	247 (99.6)	1 (0.4)	99.8 %	0.996
	Negative	247	0	247 (100)		
1/160	Positive	246	246 (100)	0	99.8 %	0.996
	Negative	249	1 (0.4)	248 (99.6)		
1/640	Positive	244	244 (100)	0	99.4 %	0.988
	Negative	251	3 (1.2)	248 (98.8)		
1/2560	Positive	240	240 (100)	0	98.6 %	0.972
	Negative	255	7 (2.8)	248 (97.3)		
1/10,240	Positive	221	221 (100)	0	94.8 %	0.895
	Negative	274	26 (9.5)	248 (90.5)		
TOTAL		495	247 (49.9)	248 (50.1)		

*All p values for the kappa statistics are <0.001

**Table 4:** Performance characteristics of the KHB/STAT-PAK®/Unigold™ tiebreaker algorithm with dilution as a supplementary test compared to gold standard (N = 2895)

Dilution	Sensitivity (95 % CI)	Specificity (95 % CI)	PPV (95 % CI)	NPV (95 % CI)
1/10	100 % (95.8-100)	99.9 % (99.6-100)	98.4 % (96.0-99.6)	100 % (98.5-100)
1/40	100 % (98.5-100)	100 % (99.8-100)	99.6 % (97.8-100)	100 % (98.5-100)
1/160	99.6 % (97.8-100)	100 % (98.5-100)	100 % (98.5-100)	100 % (99.8- 100)
1/640	98.8 % (96.5-99.8)	100 % (98.5-100)	100 % (98.5-100)	99.9 % (99.7-100)
1/2560	97.2 % (94.3-98.9)	100 % (98.5-100)	100 % (98.5-100)	99.7 % (99.5-99.9)
1/10,240	89.5 % (85.0-93.0)	100 % (98.5-100)	100 % (98.3-100)	99.0 % (98.6-99.4)

### Results on discordant RDTs

The serial algorithm KHB/STAT-PAK® had 24 discordant results, all of which were resolved negative. The 1/40 strength misidentified one discordant as positive whereas the 1/10 dilution had 3 false positives. The higher dilution strengths from 1/160 and up were able to correctly identify all 24 as negative. An alternate serial KHB/Unigold™ algorithm had 9 discordant results, 2 of which were positive on gold standard. All dilution strengths correctly identified all 9 discordants.

## Discussion

The dilution test was able to correctly identify all 18 false positive results at a level of 1/160 with a specificity of 100 %. However the improved specificity came at a cost of sensitivity, as the sensitivity worsened with increasing dilutions.

To further understand the sensitivity of the new methodology, we examined the false negative results at lower dilutions to determine if they were more likely to be due to early seroconversion or to poor sensitivity of the dilution test. One gold standard positive sample tested positive at a dilution of 1/40 but negative at 1/160. This sample had a weakly positive test line on all 3 RDTs, an indeterminate result on WB (+gp160, +p24) and a positive result on PCR. This individual was tested due to presence of a sexually transmitted infection and listed the most recent exposure as 19 days prior to testing. CD4 was 428. This sample was suggestive of seroconversion. There were 2 additional false negative results at the dilution level of 1/640. One was positive on KHB and Unigold™, weakly reactive for STAT-PAK®, and positive on WB. Reason for testing was risk of exposure with most recent exposure 11 days prior to testing. CD4 was 260. The second sample was positive on all 3 RDTs, WB and PCR. Reason for testing was pre-marriage screening and no exposure risk was listed. CD4 was 647. Both of these samples were unlikely to be seroconversion. More data is needed, however this suggests that occasional true positive samples with established HIV infection will be misclassified by dilution. This is relevant, as instructing the patient to return to be re-tested after several weeks as is done now when early seroconversion is suspected, may not correctly resolve these false negatives.

Given the risk of false negative results with the dilution test, we conclude that the dilution test cannot replace WB as a confirmation test. However its ability to differentiate false positives from true positives, suggests that it has potential to be used as a supplementary test in a confirmatory algorithm where RDT algorithm positive samples are confirmed with dilution, and negative dilution samples are referred to a higher-level laboratory for NAT. The referral for NAT is facilitated by the recently increased availability of PCR testing on dried blood spots for infant testing.

### Comparison with existing supplementary tests

The performance of dilution as a supplementary test compares well to published evaluations of both Multi-spot and Geenius. An American study of Multispot in an algorithm with Architect 4^th^ generation immunoassay showed that Multispot identified a similar proportion of true positives as Western Blot, 93.7 % versus 94.4 % [13]. There were 90 (13.8 %) samples that were positive on the screening assay and negative on Multispot. These were resolved by NAT, to reveal 47.8 % were false positives on the screening assay and 52.2 % were early seroconversion. A Canadian study compared Multispot with Geenius on a panel of samples [14]. Sensitivity for both was 100 %, however specificity was 99.1 % for Multi-spot and 96.3 % for Geenius (p = 0.688). Kappa value for agreement between the two tests was 0.96 (0.92-0.99).

An ideal confirmation test for resource limited settings would be low cost, simple and rapid, robust in terms of temperature and environmental conditions, with both high sensitivity and specificity and few indeterminate results. The dilution test uses RDTs that are commonly available in sites doing HIV testing. It requires less skill than the WB, and is similar in skill level to that of the OIC as it also requires precise pipetting techniques. In most cases, only 2 dilutions need to be tested, giving a cost of approximately $3 USD and a turnaround time of 30–40 minutes. This compares to 2 hours for the OIC, 15 minutes for the Multi-spot and 3 hours for WB. An advantage is that there are no indeterminate results, as with OIC, Multi-spot and WB. Indeterminate results are undesirable, as they require individuals to come back for further testing with the attendant risk of failure to follow up. The dilution test can not be used to definitively rule out HIV infection due to the risk of false negatives and requires all negative results to be tested with NAT. However for the majority of screen positive samples, same day results would be available. In our cohort, using the tiebreaker algorithm presently in use in Ethiopia, this would mean 246 of 265 (92.8 %) screen positives could be immediately confirmed at a dilution of 1/160, while 19 would go on to further testing to ultimately identify 18 false positives. In other words, one positive sample would be unnecessarily subjected to an additional test. A dilution of 1/640 would result in 244/265 (92.1 %) positives immediately confirmed, and 21 sent for NAT.

The 2013 US diagnostic algorithm emphasizes the importance of detection of acute HIV infection, and discrimination between HIV 1 & 2. Dilution testing used as part of a supplementary testing algorithm with NAT, would identify early seroconversion. Discrimination between HIV 1 & 2 would not be addressed aside from that gleaned from discriminatory HIV RDTs. This is a similar limitation to Western Blot, which is known to be inaccurate at discriminating between HIV 1 & 2.

### Theoretical basis for dilution methodology

Our methodology is based on the principles used to identify recent HIV infection for the purposes of incidence surveys. Here recent infection versus established infection is distinguished through an algorithm whereby samples are tested for HIV with enzyme immunoassays (EIA) with high sensitivity. Positive samples then go on to get a second, less sensitive test. The less sensitive test is an EIA test that is modified by use of a diluted sample and reduced incubation time. Samples positive on both tests are determined to be established infection, while those positive on the sensitive (S) test but negative on the less sensitive (LS) test are designated recent infection. This S/LS methodology is based on the principle that antibody titres increase over a period of several months after initial infection. Samples with low antibody titres test negative on EIA when the sample is diluted, whereas samples with higher titres are persistently positive [15]. Following on the work of Constantine [16], Soroka et al. proposed a variation for resource limited settings, whereby RDTs are substituted for the EIA in the S/LS algorithm [17]. Here dilution is again used to alter the sensitivity of the RDTs. A positive result on the first RDT screening test, followed by a negative RDT result on the diluted sample suggests recent infection. This algorithm has been successfully employed in incidence surveys using different RDTs and at differing dilution levels [18, 19].

Broad spectrum antibodies are produced in the early immune response to infectious disease antigens and can cause non-specific cross reactivity in serological testing [6]. These antibodies are expected to have low avidity, as has been demonstrated by work in blood donors [20]. In proposing dilution as a methodology to confirm HIV infection, we postulate that dilution will distinguish both the low avidity antibodies, as well as the possibly low titres [21] of the cross-reacting antibodies responsible for false reactivity. We found data to support this application of dilution testing in a report from Ghana using Soroka’s methodology to identify incident HIV infection that describes how acute seroconversion was confused with false reactivity [22]. 76 samples repeatedly positive on Genescreen Ultra HIV Ag-Ab ELISA were tested on Determine™ with the S/LS methodology. 41 were positive on the sensitive RDT, and of these 18 were positive on dilution, while 23 were negative on dilution. Confirmation with INNO-LIA™ revealed that all 35 Determine™ non-reactive results were true negatives, and only 1 of 23 samples negative on dilution was HIV positive. All those positive on dilution were true positives, suggesting that dilution was able to distinguish the cross reactivity from true positivity.

### Choice of RDT

We chose to use Determine™ as the RDT as it has high sensitivity and is in common use in many countries. We also had experience with piloting dilution testing on Determine™ in the Democratic Republic of Congo and therefore had programmatic data on its performance. We diluted samples with normal saline to 1/5000 and used the OIC confirmation test as the gold standard. The proportion of agreement with OIC for 263 samples positive on both Determine™ and Unigold™ was 89.2 % (kappa 0.423) when indeterminates on OIC were excluded. The dilution test identified all 23 RDT algorithm false positives, and introduced 5 false negatives. A limitation is that no NAT was done, so it is possible that some of the false positives identified on gold standard were in fact early seroconversion.

### Use of dilution with RDT discordant samples

Finally, an unexpected finding in our study was the ability of dilution testing at a level of 1/160 to correctly resolve all 33 RDT discordant results from the serial algorithms in our study. Discordant results occur when one RDT is positive and the other negative on a serial or parallel algorithm. They are undesirable because they result in an inconclusive result for the client that requires re-testing in two weeks [1]. National programmes often use a tiebreaker algorithm to avoid these inconclusive results with their attendant risk of loss to follow up despite the known high risk of false positives results with the tiebreaker algorithm [2, 9, 23, 24]. Our results suggest that dilution testing has the potential to immediately confirm positive results and to allow a preliminary negative result to be given to clients with discordant RDT results. Discordant samples negative on dilution would still require follow up testing in two weeks and/or referral for NAT. Further formal study is needed to explore this potential role for dilution testing in resolving discordant RDT results.

### Limitations

The strength of our study is that NAT testing was available to resolve indeterminate WB samples which made it possible to rule out early seroconversion as a potential cause of false positive results. It is limited by its application in a single cohort in Ethiopia with a single RDT. More work is needed to determine whether our results are replicable in other locations and with other RDTs. In addition, each site using dilution as a supplementary test would need to establish an appropriate dilution cut-off prior to using the test. The methodology would be similar to what has been used in incidence surveys [17].

## Conclusion

Our study is a first step in providing proof of concept for a new, cost effective method of confirming HIV diagnosis in resource-limited settings. It has potential for use as a supplementary test in a confirmatory algorithm, whereby double positive RDT results are tested by dilution, with positive results confirming HIV infection. Negative dilution results would require NAT testing to rule out false negative results either due to seroconversion or misclassification by the lower sensitivity dilution test. Further research is needed to determine if our results can be replicated in other settings.
